# Supplementary material for: Causal Relationship between Aging and Anorexia Nervosa: A White-Matter-Microstructure-Mediated Mendelian Randomization Analysis
Source: Biomedicines. 2024 Aug 16;12(8):1874. doi: 10.3390/biomedicines12081874 (PMC11351342; doi:10.3390/biomedicines12081874)
Supplement: Supplementary file 1 [file biomedicines-12-01874-s001.zip › Supplementary Figure/Figure S8/Leaveoneout plot of MR analysis for TL to ubm-b-2093.pdf]

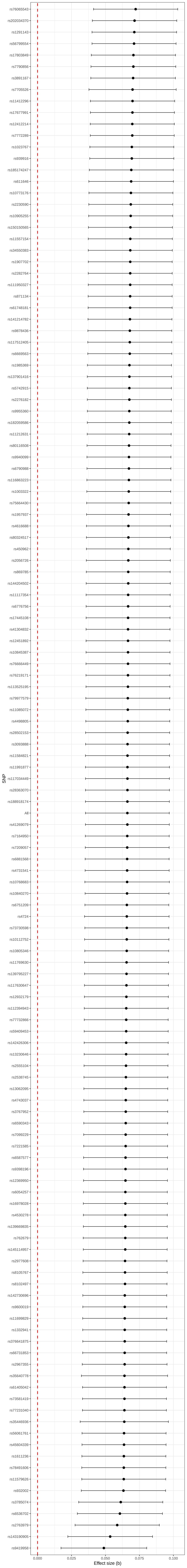
rs76065543
rs202034370
rs1291143
rs56799554
rs17803849
rs7790856
rs3891167
rs7705526
rs11412296
rs17677991
rs12412214
rs7772289
rs1023767
rs939916
rs185174247
rs611646
rs10773176
rs2230590
rs10905255
rs150150565
rs11557154
rs34550383
rs1907702
rs2282764
rs111950327
rs871134
rs61748181
rs141214782
rs9878436
rs117512405
rs6669563
rs1985369
rs137901416
rs5742915
rs2276182
rs9955360
rs182059586
rs11212631
rs80116508
rs9940099
rs6790988
rs116863223
rs1003322
rs75664430
rs1957937
rs4616688
rs80324517
rs450962
rs2056726
rs869785
rs144204502
rs11117354
rs6776756
rs17445108
rs41304832
rs12451892
rs10845387
rs76666449
rs76219171
rs113525195
rs79977579
rs11085072
rs4498805
rs28502153
rs3093888
rs11584821
rs11991877
rs117034449
rs28363070
rs188918174
All
rs41269079
rs7164950
rs7209057
rs6881568
rs4731541
rs10768683
rs10840270
rs6751209
rs4724
rs73730598
rs10112752
rs10805346
rs11769630
rs139795227
rs117630647
rs12932179
rs112394943
rs77732866
rs59409453
rs142426306
rs13230646
rs2555104
rs2538745
rs13062095
rs4743037
rs3767952
rs6590343
rs7099229
rs7221585
rs6587577
rs9398196
rs12369950
rs6054257
rs16978028
rs4530278
rs139669835
rs762679
rs145114957
rs2977608
rs8105767
rs8102497
rs142730696
rs9600019
rs11699829
rs1332941
rs376641875
rs66731853
rs2967355
rs35640778
rs61405042
rs73581419
rs77231040
rs35446936
rs56061761
rs45604339
rs1611236
rs78491606
rs11579626
rs932002
rs3785074
rs6536702
rs2763979
rs143190905
rs9419958
SNP
0.000
0.025
0.050
0.075
0.100
Effect size (b)
